# Beyond a negative trial: timing, molecular residual disease, and antibody biology in adjuvant immunotherapy for resected NSCLC

**DOI:** 10.3389/fonc.2026.1941683

**Published:** 2026-09-10

**Authors:** Satı Coşkun Yazgan, Elif Berna Köksoy, Hakan Akbulut

**Affiliations:** Ankara University School of Medicine, Department of Medical Oncology, Ankara, Türkiye

**Keywords:** adjuvant immunotherapy, circulating tumor DNA, molecular residual disease, nivolumab, non-small cell lung cancer

## Abstract

The phase 3 EA5142/ALCHEMIST study of adjuvant nivolumab for completely resected EGFR/ALK-negative non–small cell lung cancer (NSCLC) is being widely interpreted as a negative trial. Its immediate clinical significance is that nivolumab monotherapy, given after the completion of standard postoperative treatment, did not offer a disease-free survival advantage in this population. However, inconsistent results from adjuvant, neoadjuvant, and perioperative trials raise unanswered questions about treatment sequencing, residual disease burden, biomarker selection, and trial design. We propose a hypothesis-generating timing × molecular residual disease (MRD) framework to organize these questions, while recognizing that cross-trial comparisons cannot establish either treatment timing or MRD status as a causal determinant of efficacy. NADIM-Adjuvant is the first trial to demonstrate that postoperative circulating tumor DNA positivity can serve as a prognostic marker; however, these data do not confirm MRD as a predictive biomarker for response to chemoimmunotherapy. We also assess an antibody-biology hypothesis mediated by the interplay between immunoglobulin G subclasses and Fcγ receptors that may drive inter-agent heterogeneity, although this has not been clinically established in resected NSCLC. These considerations collectively identify several testable hypotheses for future adjuvant trials, without diminishing the negative clinical outcome of EA5142.

## Introduction

Adjuvant immunotherapy has substantially reshaped the management of resected NSCLC, yet the underlying evidence base remains notably heterogeneous. Atezolizumab administered after platinum-based chemotherapy improved disease-free survival (DFS) in IMpower010, with the most pronounced signal observed in stage II–IIIA disease and in tumors expressing PD-L1 ≥1% (1). In PEARLS/KEYNOTE-091, pembrolizumab likewise improved DFS in the overall resected population, although the magnitude of benefit across PD-L1 subgroups proved less consistent (2). Against this background, EA5142/ALCHEMIST demonstrated no DFS advantage with nivolumab relative to observation, including among patients with high PD-L1 expression (3). The differences among the major adjuvant trials raise broader questions about treatment sequencing, residual-disease burden, and biomarker selection that merit prospective investigation.

IMpower010, KEYNOTE-091 and EA5142 are the main randomized adjuvant studies that inform adjuvant checkpoint-inhibitor monotherapy postoperatively, while NADIM-Adjuvant provides complementary evidence by evaluating concurrent with postoperative chemotherapy and maintenance nivolumab (1–4). Randomized neoadjuvant and perioperative studies provide a perspective on the potential relevance of initiating immunotherapy earlier in the course of treatment, albeit not one directly comparable to these trials (5–8).

## Reframing discordant adjuvant immunotherapy results

One cannot simplify these different results into a comparison between nivolumab, pembrolizumab, and atezolizumab. Instead, they may simply reflect clinically and biologically heterogeneous contexts in which checkpoint inhibition was given, at least in part. In EA5142, nivolumab could be started quite late; randomization was allowed up to 8 months post-surgery for patients receiving adjuvant chemotherapy and up to 10 months post-surgery for those receiving chemotherapy plus radiotherapy (3). This time interval could distinguish checkpoint inhibition from antigen release and immune activation due to chemotherapy. But because EA5142 did not randomize patients with respect to the timing of surgery, chemotherapy, or immunotherapy, the trial is unable to evaluate treatment timing as a factor contributing to the lack of benefit.

Substantial differences in eligibility criteria, disease stage, PD-L1 assays and subgroup definitions, chemotherapy requirements and regimens, postoperative radiotherapy use, control groups, endpoint definitions, and follow-up duration and statistical maturity between trials further complicate interpretation (1–4). IMpower010 mandated cisplatin-based adjuvant chemotherapy before randomization and compared atezolizumab with best supportive care, while KEYNOTE-091 randomly assigned pembrolizumab versus placebo after complete resection, having strongly recommended but not universally mandated the induction of adjuvant chemotherapy (1, 2). While EA5142 assessed nivolumab following indicated postoperative treatment, NADIM-Adjuvant combined nivolumab and postoperative chemotherapy concurrently before continuing it as maintenance therapy (3, 4). Such differences render it impossible to solely ascribe the disparate outcomes to timing, MRD status, or antibody selection.

## Timing in relation to chemotherapy-induced immune priming

NADIM-Adjuvant was the first randomized phase 3 trial to report adjuvant chemoimmunotherapy in resected NSCLC, enrolling 206 patients who received adjuvant carboplatin/paclitaxel with or without concurrent and maintenance nivolumab. With a median follow-up of 34 months, the 3-year relapse rate was lower with chemoimmunotherapy (26.7% vs 40.1%), and a prespecified cancer-specific DFS analysis favored the experimental arm (4). Although the main DFS boundary was not crossed, they are limited by immature follow-up and being available so far only as an early conference report. Thus, the numerical difference does not establish that concurrent nivolumab and chemotherapy is superior to a delayed surgical approach. Still, it provides a clinically relevant basis for a prospective study of whether the order of treatment affects efficacy.

The timing hypothesis also has a wider clinical context. Neoadjuvant nivolumab plus chemotherapy compared with chemotherapy alone. Significant improvements in event-free survival and pathological complete response were shown by CheckMate 816 (5). Perioperative pembrolizumab, durvalumab, and nivolumab regimens subsequently improved event-free survival in KEYNOTE-671, AEGEAN, and CheckMate 77T, respectively (6–8). These findings show that checkpoint-inhibitor–containing regimens can exert clinically meaningful effects when administered before surgery, with the primary tumor and its antigenic repertoire intact. But these studies do not isolate timing as the determinant of benefit, because they differ from adjuvant-only trials in disease setting, chemotherapy backbone, duration of therapy, endpoint definitions, and whether preoperative or postoperative treatment was administered. However, taken together, the evidence indicates that evaluation of treatment timing should be performed prospectively, but it does not prove that earlier delivery explains the negative result we observed with EA5142.

## Antibody biology as a potential modifier of efficacy

Explaining the inter-agent heterogeneity, a complementary explanation could lie in the structure of the antibody itself. The immunoglobulin G subclasses and Fc engineering of anti-PD-1 and anti–PD-L1 agents differ significantly, which alters binding to all human immune cell types expressing Fcγ receptors, antibody-dependent effector functions, and the fate of PD-1-expressing T cells (9, 10). Yet such mechanistic differences will not always translate into clinical differences between available agents. Both nivolumab and pembrolizumab are anti–PD-1 antibodies; thus the failure of their trials is not attributable only to an anti–PD-1 versus anti–PD-L1 distinction. Biologically plausible modifiers that differ between these antibodies include Fc structure, epitope recognition, binding kinetics, receptor occupancy, dosing, and pharmacokinetics; however, no single factor has been demonstrated to explain the differences between the results of EA5142 and KEYNOTE-091.

Preclinical studies demonstrate that Fc-mediated depletion or modulation of PD-1–expressing immune cells can influence checkpoint-antibody activity, but it remains unclear whether these mechanisms are important for antitumor immunity in the postoperative setting in micrometastatic NSCLC. In this setting, the lack of an adequately powered head-to-head randomized comparison of individual PD-1 or PD-L1 antibodies gives no firm conclusion, and there is currently no validated Fcγ receptor biomarker linking Fcγ receptor biology to adjuvant efficacy. Overall, antibody biology remains a mechanistic hypothesis rather than an accepted clinical explanation for intertrial heterogeneity.

## Patient selection and the limits of PD-L1

PD-L1 immunohistochemistry is still an imperfect adjuvant biomarker. Its predictive value varies widely between IMpower010 and KEYNOTE-091, and in EA5142, even patients with high PD-L1 expression derived no benefit (1–3). These heterogeneous results support exploring additional biomarkers but do not prove that a disease-burden marker could supersede PD-L1. Other sources of variability, such as differences in the assays used, patient populations treated, and treatment designs, could also underlie the apparent discordance between PD-L1 expression and clinical benefit.

## Lessons from urothelial cancer

In this regard, urothelial experience is informative. In the unselected IMvigor010 population, adjuvant atezolizumab was not associated with benefit in urothelial carcinoma, a distinct disease from NSCLC, but a clear benefit was observed in a ctDNA-defined MRD-positive subgroup, which showed an exploratory treatment-effect signal; this observation informed the design of the MRD-guided IMvigor011 (11, 12). The randomized evidence for ctDNA-guided treatment in muscle-invasive urothelial cancer subsequently came from the later IMvigor011 trial (12). However, differences in tumor biology, ctDNA shedding, systemic therapy, and patterns of recurrence preclude direct extrapolation to NSCLC. The urothelial experience therefore demonstrates the feasibility of an MRD-guided trial strategy, rather than proving that MRD selection will identify patients who will benefit from adjuvant immunotherapy in resected NSCLC.

## Molecular residual disease as a candidate axis

Postoperative ctDNA is a strong candidate for identifying patients with an increased risk of recurrence but cannot reliably exclude residual disease in the absence of disease. In particular, ctDNA-based MRD evaluation was performed prospectively in the NADIM-Adjuvant trial and showed that postoperative MRD positivity was associated with poorer DFS (HR 5.7; 95% CI 1.0–31.2; p=0.045) (4). This association supports the prognostic relevance of postoperative MRD in the reported dataset, although the wide confidence interval, limited number of events, and immature follow-up require caution. Importantly, prognostic association does not establish predictive utility. The data presented do not provide evidence that MRD-positive patients receive relatively greater benefit from nivolumab-containing therapy, nor that treatment can be safely omitted in MRD-negative patients. That would necessitate an *a priori* determination of whether the treatment effect is different by MRD status.

## Practical and methodological limitations of ctDNA

Analytical and biological heterogeneity pose significant obstacles to the clinical utility of post-operative ctDNA measurements. Assays differ in sequencing depth, error-suppression methods, genomic coverage, bioinformatic thresholds, and definitions of MRD positivity. While tumor-informed platforms identify patient-specific variants and can be highly specific, they require sufficient tumor tissue to create a personalized assay, necessitating extensive development time and additional turnaround time. While tumor-agnostic approaches can be more easily deployed, they may be less sensitive to patient-specific changes and more subject to background signals, including those arising from clonal hematopoiesis (13).

Sensitivity is especially problematic in patients who have low-volume residual disease or tumors with minimal ctDNA shedding. A negative postoperative result could be due to undetectable shedding, low plasma volume, inadequate sampling timing, or detection at the limit of analysis rather than the absence of disease. Surgery-mediated elevations in non-tumor cfDNA could hamper testing at very early time points, and postponing sampling might delay adjuvant therapy. While longitudinal assessment may increase sensitivity, it is more costly and logistically complicated; furthermore, there is no clear consensus on how best to interpret transient vs. persistent ctDNA positivity (13).

Detection at an earlier stage is also influenced by lead-time bias, and its associated fate does not improve survival unless a valid therapeutic intervention exists. Therefore, an MRD strategy should demonstrate clinical value by translating into better patient outcomes rather than merely being detectable compared with histopathology. Other barriers to more widespread implementation include cost, reimbursement, sample requirements, turnaround time, access to appropriate reference laboratories, and interlaboratory comparability. Until clinical utility is established by randomized trials, ctDNA-guided escalation or de-escalation of adjuvant immunotherapy should remain investigational.

## A hypothesis-generating timing × MRD framework

**Figure 1:**
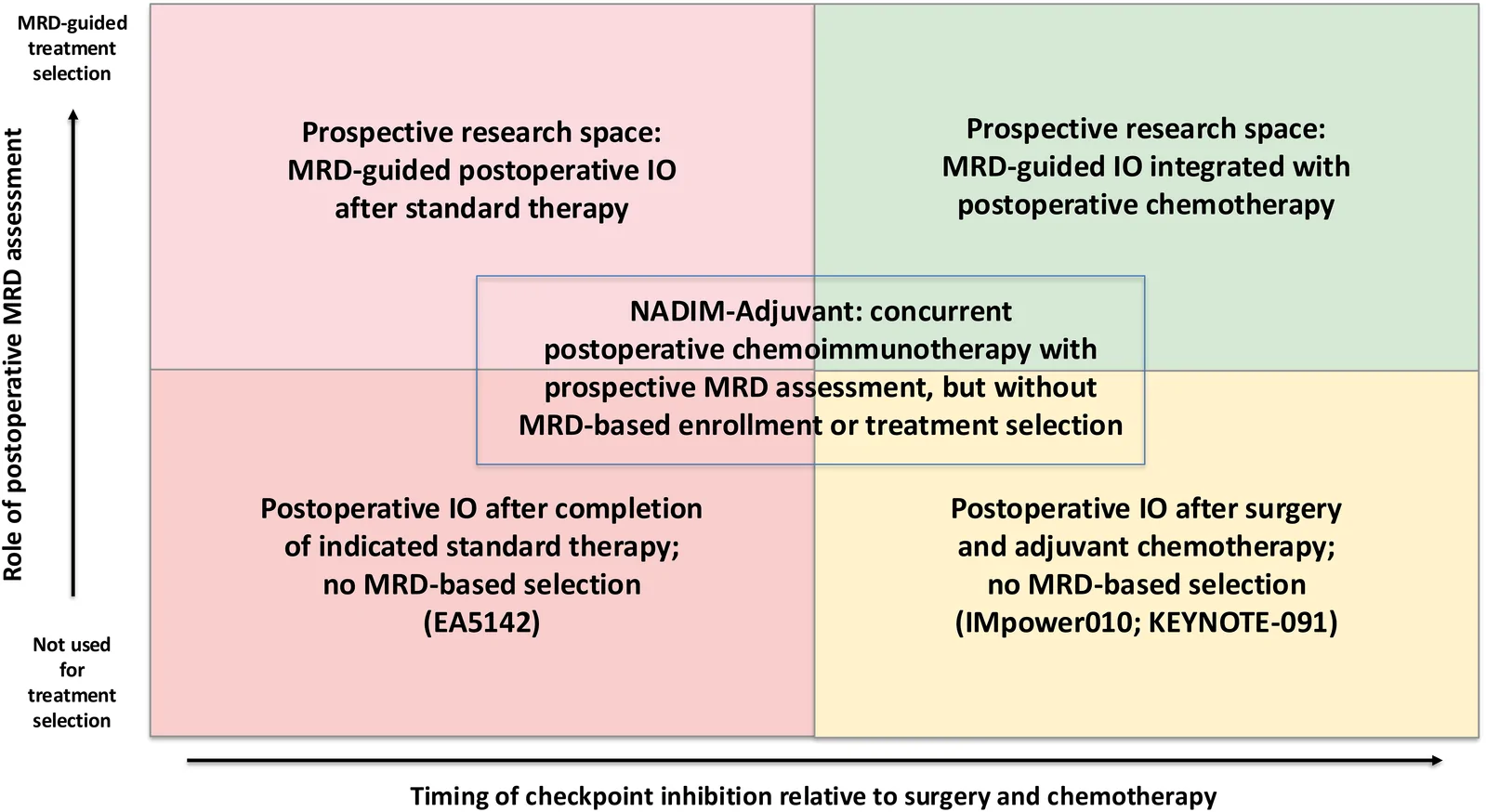
Hypothesis-generating timing x MRD framework for immunotherapy trial design in resected NSCLC. IO, immunotherapy; MRD, molecular residual disease; NSCLC, non-small cell lung cancer.

This conceptual framework for future adjuvant research includes treatment timing and postoperative MRD (**Figure 1**). The timing dimension captures the relationship between checkpoint inhibition and surgery and chemotherapy, while the MRD dimension captures the extent to which postoperative molecular findings inform treatment selection. Rather than classifying trials by presumed biological efficacy, the framework outlines design characteristics that could be evaluated prospectively.

Within this framework, NADIM-Adjuvant represents concurrent postoperative chemoimmunotherapy with prospective MRD assessment, but not MRD-directed enrollment or treatment (4). IMpower010 and KEYNOTE-091 represent checkpoint inhibition administered postoperatively after surgery and adjuvant chemotherapy, without MRD-based selection (1, 2). EA5142 represents nivolumab administered after completion of indicated standard postoperative therapy, also without MRD-based selection (3). Consequently, MRD-guided treatment strategies occupy a prospective research space that has not yet been validated in resected NSCLC.

## Discussion

EA5142 presents direct randomized evidence that nivolumab, administered after completion of the recommended standard postoperative treatment, did not improve DFS compared with observation in this population (3). The main clinical implication of the trial is the lack of any benefit of nivolumab compared with standard of care, including in the high PD-L1 subgroup. While this does not prove that nivolumab is intrinsically inferior to other checkpoint inhibitors, it does not support the delayed adjuvant nivolumab strategy tested in EA5142.

The NADIM-Adjuvant offers promising early insights into treatment sequencing and risk of recurrence following surgery. The numerical efficiency signal remains hypothesis-generating because it has not crossed the pre-specified DFS boundary in the context of a relatively immature follow-up. Although analysis of its ctDNA does support MRD as a prognostic biomarker, it does not demonstrate that MRD is a predictor of benefit with surgery followed by adjuvant chemoimmunotherapy (4).

Favorable results of randomized neoadjuvant and perioperative trials add to the complementary evidence that an early course with immunotherapy-containing regimens can improve outcomes but do not disentangle timing as the causal predictor of efficacy (5–8). Similarly, the urothelial IMvigor010 and IMvigor011 experience demonstrates the feasibility of MRD-guided trial design but cannot establish its clinical utility in NSCLC (11, 12).

Standardized postoperative ctDNA assays, serial sampling, and prespecified treatment-by-MRD interaction analyses can facilitate future adjuvant studies. MRD-positive escalation and MRD-negative de-escalation are separate clinical queries that need to be tested in suitably powered randomized trials. Such trials that assess timing would ideally have only minimal differences in the checkpoint inhibitor, chemotherapy backbone, eligibility criteria, and treatment duration across groups. These efforts may be supplemented by translational analyses of how Fcγ receptor engagement influences the overall response to anti-PD-1 antibody, though structure does not currently provide a way to rationally select clinical agents.

EA5142 therefore defines the limitations of the specific postoperative nivolumab strategy it tested and identifies unresolved questions. The proposed timing × MRD framework is intended to organize these questions and facilitate prospective testing, rather than to provide a validated explanation for discordant trial outcomes.

## Data Availability

The original contributions presented in the study are included in the article/supplementary material, further inquiries can be directed to the corresponding author/s.
